# Cancer and COVID-19: ethical issues concerning the use of telemedicine during the pandemic

**DOI:** 10.1186/s12913-022-08097-w

**Published:** 2022-05-25

**Authors:** Lucas Huret, Henri-Corto Stoeklé, Asmahane Benmaziane, Philippe Beuzeboc, Christian Hervé

**Affiliations:** 1 ESME Sudria, Ivry-Sur-Seine, France; 2 Department of Ethics and Scientific Integrity, Foch Hospital, Suresnes, France; 3 Department of Oncology and Supportive Care, Foch Hospital, Suresnes, France; 4 Medical School, Paris Cité University, Paris, France; 5 Medical School, Versailles-Saint-Quentin-en-Yvelines University (UVSQ), Montigny le Bretonneux, France; 6 Veterinary Academy of France, Paris, France; 7 International Academy of Medical Ethics and Public Health, Paris Cité University, Paris, France

**Keywords:** Cancer, Healthcare, Telemedicine, COVID-19, Pandemic, Ethics

## Abstract

The lockdown imposed in France during the first wave of the COVID-19 pandemic wreaked havoc with access to healthcare. From March 2020 onwards, the oncologists of Foch Hospital, like many others at hospitals throughout the world, were obliged to adapt to the new conditions, including, in particular, the impossibility of seeing patients in classic consultations for the diagnosis and treatment of cancer. Patients with cancer are particularly susceptible to this new virus, due to their immune status, and this made it difficult to carry out standard hospital visits for these patients. Some patients refused to come to the hospital, whereas the doctors decided, for others, that consultation conditions at the hospital were not sufficiently safe, with sanitary measures that had yet to be precisely defined. Telemedicine was one of the adaptations adopted during this period. This mode of consultation was little used before the pandemic, for various reasons, and reimbursement was not automatic. This new approach proved to have limitations as well as advantages, as demonstrated by our empirical ethics research study, a retrospective qualitative survey of the doctors of the oncology and supportive care departments of Foch Hospital, performed during July 2021. The interview grid was based on the studies on telemedicine, oncology, COVID-19 and empirical ethics available at the time. Based on the experience gained in this domain during the first wave of the epidemic, which hit France between March and June 2020, we identified three eligibility criteria for consultations in telemedicine: the consultation concerned should not be the first consultation, the patient should be a known patient that the doctor trusts not to minimize the description of symptoms, and the results of the patient’s evaluations and examinations must be good. It may be appropriate to continue the use of teleconsultation in the future, provided that these criteria are respected.

## Background

Telemedicine is a medical tool that has emerged from the new information and communication technologies (ICTs) [1]. The French High Authority for Health has defined it as “*a form of medical practice at distance based on the use of information and communication technologies. Its aim is to improve the accessibility of healthcare services (particularly in areas in which access is poor) and the quality of life of patients, by permitting management and follow-up at their homes.” * (https://www.has-sante.fr/jcms/c_2673715/fr/telemedecine).

France perceived the utility of such approaches during the first wave of the COVID-19 epidemic, which hit the country between March and June 2020 [2, 3]. In this context, as shown by the study on “warning and surveillance indicators for COVID-19”, many hospitals were faced with problems of saturation, with too few beds available, and questions were raised about the risks of patients coming to the hospital for consultations, which could potentially lead to their contamination [4]. Clinical practices therefore had to be rapidly adapted, particularly in zones with a high population density, such as the Parisian region, where high rates of viral spread that would be difficult to control were feared [5]. The priority therefore was — and still is — to protect patients, particularly those most vulnerable to this new virus, including cancer patients [6]. More appropriate patient management methods for this exceptional crisis situation had to be found.

One of the solutions identified was telemedicine, with virtual consultations, providing patients with access to a healthcare professional without the need to travel to the hospital [7]. This was the approach adopted by the oncology and supportive care departments at Foch Hospital, initially at the initiative of the doctors, who considered it too risky for certain patients to come to the hospital. This approach was then supported by hospital management, which supplied the necessary equipment to ensure that telemedicine could be practiced in the best possible conditions. After the use of telemedicine during the first wave of the pandemic, a teleconsultation committee was established at Foch Hospital, the role of which will be detailed below (in the results).

Given that such methods are already being proposed, the objective of this article is to study the potential ethical issues associated with their use raised by the concerns or opinions voiced by physicians. These issues should also be taken into account by the scientists and engineers responsible for developing these new ICTs. We, therefore, performed a retrospective qualitative study with the doctors of the oncology and supportive care departments of Foch Hospital, in France, who had gained experience in these practices during the first wave of the epidemic, as a means of identifying and resolving these ethical issues in the context of cancer patient management.

This study was subject to several methodological limitations, due to the small number of doctors questioned. Furthermore, as it was performed at a single hospital, it can in no way be considered representative of the entire French oncological environment. There are also other well-known limitations to this type of method [8]. Nevertheless, we believe that our results remain relevant, and that we have taken these methodological limitations into account.

## Methods

We constructed an interview grid based on previously collected elements. We chose to use a qualitative method, based on semi-directed interviews with doctors from the oncology and supportive care departments of Foch Hospital who had used teleconsultation during the first wave of COVID-19. The study was approved by the institutional review board (IRB) of Foch Hospital (00012437). Oral informed consent was obtained from all participants, and the consent form was also approved by the same IRB. All methods were performed in accordance with the relevant guidelines and regulations in France ([9], https://solidarites-sante.gouv.fr/systeme-de-sante-et-medicosocial/recherche-et-innovation/recherches-impliquant-la-personnehumaine/).

Eight doctors from the oncology and supportive care departments who had used telemedicine were interviewed with a specific interview grid (Table 1) during July 2021. The grid was based on published findings on telemedicine, cancer, COVID-19 and empirical ethics available at the time via PubMed, Cairn, Google Scholar or Google. These doctors included one oncologist who joined the department after the first wave of the epidemic and who had practiced in a region less severely hit by this first wave. This doctor was included for the purposes of comparison. The sample included both male and female doctors, of various ages and specialties (in terms of the affected organs), practicing either medical oncology or supportive care (Table 2).

**Table 1:** Grid for semi-directed interviews

1) Did you use teleconsultation during the COVID-19 pandemic?	
2) What did you think of telemedicine before the COVID-19 pandemic?	
3) Did you obtain informed consent from the patient? How did you do so? (Not just due to a simple fear of COVID-19 without awareness of the possible consequences of delayed treatment...)	
4) What tools did you use for teleconsultation? Have you any suggestions for their improvement? Are you aware of other tools used in this practice?	
5) Did you encounter any technical difficulties?	
6) Did you have to deal with any patients refusing to attend a consultation in person?	
7) What are the principal difficulties encountered in establishing a correct diagnosis via teleconsultation?	
8) Did any of your patients not have access to the necessary computing tools for teleconsultation?	
9) Did you have any contact with a third party (present with the patient) during a teleconsultation, with the explicit consent of the patient?	
10) Did the patients mention any problems with telemedicine?	
11) Did telemedicine help you in your daily practice? (diagnosis, prescription, follow-up)	
12) What impact do you feel that telemedicine had on your doctor-patient relationship?	
13) What would you say are the advantages/disadvantages/limitations of telemedicine during the COVID-19 period?	
14) Do you think that telemedicine has a future in the world of oncology, and by extrapolation, medicine?	
15) Do you intend to continue using telemedicine even once the COVID-19 pandemic is over? How do you plan to do so? (right from the first consultation, for follow-up, alternation between teleconsultations and consultations in person)	
16) What is your opinion about possible progress in telemedicine and its contribution to the medical arsenal?	
17) Do you reserve telemedicine for certain indications/diseases?	
18) Do you have anything to add that was not included in this list or that you feel is relevant?

**Table 2:** Characteristics of the doctors questioned

Sex	Age group (in years)	Number of years of practice	Specialization (organ)
M	60 – 70	> 30	Urology, Gynecology, Breast
M	50 – 60	> 20	Gynecology, Breast, ENT, Thyroid
M	40 – 50	> 20	Medical oncology
M	30 – 40	< 10	Urology
F	50 – 60	> 20	Urology
F	40 – 50	> 10	Digestive, Breast, Gynecology
F	30 – 40	< 10	Generalist
F	30 – 40	< 10	Gynecology, Breast, Neurology

The duration of the interviews varied between doctors, ranging from about 25 min to more than an hour. All the interviews took place at the hospital, during the doctors’ working hours. The interviews were recorded with an application on a Samsung smartphone. Interviews were conducted face-to-face by Mr. Lucas Huret, a biomedical engineering student and intern in the Department of Ethics and Scientific Integrity, in collaboration with the Department of Oncology and Supportive Care.

The recordings were effaced after their anonymized transcription, exclusively in French, into Word on the professional computer of Mr. Lucas Huret. The raw data were not translated into English. These interviews constituted the study material for this investigation, and we analyzed their content, without the assistance of specific software. We performed an analysis of content for the “manual” extraction of pertinent information. Various excerpts are presented in the results and discussion, in italic typescript, to highlight particular points. Saturation was achieved very rapidly for the major items, making it possible to draw a number of conclusions.

## Results

Our interviews revealed a large difference between doctors in terms of their views concerning telemedicine before and after the first wave of the COVID-19 epidemic in France. Indeed, before the first wave, several doctors had no opinion on the matter, principally because they had used telemedicine only rarely, if at all, whereas others were clearly either in favor or against the use of telemedicine. Opinions ranged from enthusiasm — as exemplified by this quote from one doctor: *“I was very enthusiastic before. I told myself ‘yes, it will be great’ because, in oncology, there are a large number of consultations that can take place as teleconsultations.” —* to *“spontaneous suspicion”* — as exemplified by the statement of another doctor: *“I was spontaneously suspicious, maybe because I am no longer…I do not belong to the younger generation and I didn’t grow up with computer tools”* in response to the second question on the interview grid. However, this difference disappeared after the first wave of the epidemic. A consensus even emerged concerning certain benefits of this tool, which we will discuss later. However, views differed as to the scale at which it could be used and the number of teleconsultations to be performed, which naturally differed between the suspected diseases. This divergence of opinion also appeared to be generational (Table 2). Indeed, doctors from younger age groups appeared to be more inclined to use teleconsultation. *“I hope, effectively, that we will use it more. It can help, yes, it’s not a ‘lighter’ consultation, because the goal is not to lighten the consultation with the doctor but to be able to propose a mode of consultation for certain types of patient.”* However, regardless of the generation to which the doctors belonged, they were unanimous on two points: (1) that they will continue to use telemedicine after the pandemic has ended and (2) that telemedicine needs a new clear and operational regulatory framework. *“The context of teleconsultation requires really good regulation”* is a typical phrase from an older oncologist whose view was subsequently revised. *“A bit like the world of business discovering working from home, we discovered teleconsultations. So, yes, I think it will remain and that, for a certain number of cases, it will be good”* said one middle-aged oncologist interviewed, who suspected that only a certain number of cases could be treated by teleconsultation.

With the perspective of such regulation, the doctors expressed six, not necessarily cumulative, conditions for selecting cancer patients for management by teleconsultation. The results (Table 3) are illustrated by the following excerpts from interviews. Firstly, three conditions for eligibility for teleconsultation were identified: (1) Teleconsultation should not be used for the first consultation. *“First consultations, I refuse to do them in teleconsultation. It is not possible not to see the patient for the first consultation”;* (2) The patient must be a known patient that the doctor trusts not to minimize the description of symptoms. *“Teleconsultations are with people you know. The ideal profile is that. I know my patients, they know me and I know that they will talk frankly about things that are wrong and not try to hide things from me”.* Indeed, *“The issue with teleconsultation is person-dependent or patient-dependent. I treat ENT cancers, that means, globally, 80% alcohol- or tobacco-dependent, with patients who are dependent on alcohol or tobacco. They aren’t people who complain easily or people who will pick up the phone to call the emergency services when something is wrong.”* The notion of “behavior” is particularly important in this last quote; (3) The patient’s evaluations and examinations must be good. *“If a biological evaluation arrives and is satisfactory, and there is a normal PET scan, effectively, we can do a teleconsultation”* Although only seven of the eight doctors raised this condition, this view was unanimous. The eighth doctor simply did not speak about it in the interview. Three other conditions influencing the choice of the doctors but that appeared secondary were also raised: (4) Patient on oral treatment. *“We can also perform check-ups for patients on oral treatments because there are a certain number of oral therapies that require blood tests to check for tolerance. In such cases, we are not obliged to see the patient physically; these are appropriate consultations.”* (5) Cured patients, during monitoring, and patients in follow-up. *“For everything that is surveillance and follow-up, in my opinion, it is really a tool that should be used”*. However, this opinion was not shared by everyone: *“What bothers me in teleconsultations for surveillance, is that I have the impression that I could miss a relapse. I am always afraid of that during teleconsultations.”*; (6) Patients unable to travel easily or living a long way away. *“I have received the scans, the assessments, and I select those to whom I can propose teleconsultation. It’s true that it also takes time. So, I have a look, and if they live a long way away, my secretary phones them […] and then we do what the patients want. If they want to come, we let them come. If they prefer, we transform the consultation into a teleconsultation.”* However, it should be pointed out that, even during the COVID-19 period, some patients eligible for teleconsultations nevertheless attended visits *“in person.”* If the doctors had a doubt or thought that teleconsultation was not appropriate, they organized appointments for consultations in person. For the doctors questioned, teleconsultation is not suitable for all patients. It is *“patient-dependent”*, an expression used in several of our interviews. It was also considered *“disease-dependent”*, albeit by only three of the eight doctors interviewed. Indeed, these doctors said: *“It’s disease-dependent, it’s specialty-dependent”, “I don’t see myself, for example, in teleconsultation with a patient with a brain tumor, particularly a glioblastoma, that’s for sure.”* Finally, and this element is fundamental, it is impossible to perform only teleconsultations. According to the doctors interviewed, teleconsultations should be used in alternation with consultations in person. *“It shouldn’t be 100% of consultations like that. That is not possible. Alternating seems like a good compromise to me.”*

**Table 3:** Classification of the conditions for eligibility for teleconsultation

Type of condition	Conditions	Number of doctors
Imperative (must be fulfilled for the patient to be considered eligible for teleconsultation)	1 - Teleconsultation should not be used for the first consultation	8 of 8
	2 - The patient must be a known patient that the doctor can trust not to minimize the description of symptoms	8 of 8
	3- The patient’s evaluations and examinations must be good	7 of 8
Secondary	4- Patient on oral treatment	4 of 8
	5 - Cured, under surveillance or in follow-up	6 of 8
	6 - Patient unable to travel or living a long way away	6 of 8

Despite this consensus, the clinicians adhered to certain principles. The first, and probably most complex concerned the problem of *“the absence of clinical examination”.* Such examinations are, for the moment, not possible in teleconsultation. The doctors explicitly said *“What is missing is the clinical examination. If someone says ‘I’m having trouble breathing’, you need to use a stethoscope to know whether it’s the heart or the lungs. And you can’t do that. It’s a limitation of the exercise.”* Thus, in teleconsultation, no palpation is possible, unless performed by the patient, with guidance from the doctor. But such palpation is of little or no utility. Teleconsultation nevertheless represents a considerable gain of time for the doctor, because the clinical examination takes up a large proportion of the consultation, between the patient having to undress, be examined and then get dressed again, as pointed out by one of the doctors questioned: *“You go faster than in consultations. They are a bit shorter, because we don’t do a physical examination.”* Teleconsultation also has non-negligible economic advantages, as indicated by one of the doctors interviewed: *“At a social level, there is no competition. Because you don’t need transport vouchers for ambulances or taxis or light medical vehicles […]. You save on all the transport costs of patients.”*

The issue of image quality during teleconsultation must also be considered, in addition to that of clinical examination. Image quality is far from poor in most cases, but may not be sufficient to identify abnormalities, or possibly may be able to identify only those that are superficial. This problem is aggravated by the lack of mobile phone network coverage in certain geographic zones, particularly in provincial areas, which may render images totally unreadable, and exchanges with patients may be incomprehensible due to the phone repeatedly cutting out, as explained by one doctor as follows: *“At the start, it was real hell. The consultation could end up being by telephone because of bugs…too weak a network signal, it cut out, we couldn’t download the documents.”* This could lead to a poor understanding of medical directives by the patient and a greater difficulty for doctors to identify elements that could lead them to choose to hold a consultation in person. In such cases, the doctors switched to telephone consultations. In particular cases, if possible, the doctor sought assistance from a third party, generally a relative of the patient, present with the patient. This situation was particularly frequent for elderly patients or foreign patients who spoke French badly or not at all.

*“ I think that the essential problem is improving network coverage*.”

*“I follow lots of women with breast cancer. I diagnose recurrences on the skin with nodules that appear, like little grains of rice, that pass unnoticed by the woman — ‘Oh yes, I’ve had that for a couple of weeks’ — like a little wart. Except that it isn’t a wart. It’s a recurrence of cancer and it comes back on the skin. And, that, if you don’t examine your patient, you don’t pick it up*.”

In addition, before a teleconsultation, the patient connects to what is known as the *“virtual waiting room”*, an expression that was also used in two of our interviews. This waiting room has the same purpose as the waiting room in which patients await their consultations in person. However, as it is virtual, the patients cannot see the doctors and, therefore, cannot know whether they are ready to talk to them. The problem is that, during the first wave of the epidemic, the doctors were overworked and, in some cases, the waiting time was sufficiently long that the patients decided to disconnect, thinking that the doctor had forgotten them. This was expressed in words by two doctors. *“I had patients that I saw late, and they told me ‘I thought that you weren’t going to phone me, that it was finished. I nearly went out to do the shopping’, and afterwards, when you phone, they aren’t there anymore, because they have gone out to do the shopping. And that happens.” “Sometimes, when we had one patient physically present and another in the virtual waiting room, we kept the one with the virtual consultation waiting, as that patient was at home. After a while, the patient disconnected and it was impossible to re-establish the connection. We had to use the telephone, etc.”* In such cases, it ends up being impossible for the doctors to inform the patients of their presence. Such things do not happen during consultations in person, at least at Foch Hospital, given that the patient sees the doctor in a consulting room. *“Relative to physical presence, where the patients can see that the doctor is in the consulting room in front of them, and they know that if someone else goes in before them, it will soon be their turn.”*

Finally, we have particular experience, thanks to one of the oncologists questioned, who is a member of the teleconsultation committee mentioned in the introduction. This committee was set up as a consequence of the increasing awareness of telemedicine resulting from its use. This oncologist explained the objectives of this committee. Its goal is *“to harmonize teleconsultation”*, in other words, to create regulations concerning the use of teleconsultation at the hospital to simplify its integration, to prevent its inappropriate use and to define good practice. These reflections on the regulation of telemedicine have culminated in a *“teleconsultation guide”*. To this end, a questionnaire on telemedicine was established, which was sent to all hospital staff. The committee then proposed to develop a teleconsultation guide for patients and doctors, which we have not yet seen. In this context, the question of the platform used for consultations was also raised. The consultation committee of Foch Hospital did not wish to continue to be dependent on the platform, particularly for the transmission of medical documents, which they felt would be too complex. This was explained by the oncologist as follows: *“Transmitting documents via this platform, it’s extremely complicated, that’s why I don’t use it at all for that. I send the patient documents by post or e-mail, or we pass them directly to the patient’s pharmacy if necessary, but I do not transmit documents, prescriptions, sick leave notes or anything else via this platform. It’s too complicated.”* Such transmissions are, thus, currently performed by e-mail or post, in line with the directives of the French Ministry of Solidarity and Health (https://solidarites-sante.gouv.fr/soins-etmaladies/maladies/maladiesinfectieuses/coronavirus/professionnels-desante/article/teleconsultation-et-covid-19-qui-peut-pratiquer-adistance-et-comment) .

## Discussion

Based on the experience gained by these doctors in this domain during the first wave of the epidemic, which hit France between March and June 2020, we identified three eligibility criteria for telemedicine consultations in oncology (Table 3): the consultation concerned should not be the first consultation, the patient should be a known patient that the doctor trusts not to minimize the description of symptoms, and the results of the patient’s evaluations and examinations must be good. It may be appropriate to continue the use of teleconsultation in the future, provided that these criteria are respected. Neverthelessseveral points merit consideration in greater detail.

As shown in the results, before COVID-19, these doctors had no real experience with telemedicine. Fortunately, the decree of August 1, 2018 in supplementary clause 6 of the medical convention, and the decree of September 13, 2018 had specifically mentioned the possibility of using telemedicine (https://www.legifrance.gouv.fr/loda/article_lc/LEGIARTI000037439234/2018-08-11, https://www.legifrance.gouv.fr/loda/id/JORFTEXT000037399738/) . The use of telemedicine was seen as a way of promoting equal access to care in remote areas, rather than as a tool for use in pandemic conditions, but the conditions were soon met. Following their use of telemedicine in the exceptional conditions of a pandemic and patient lockdown, the eight doctors questioned here considered the need to specify the conditions in which routine teleconsultation would be possible for future consultations. Our results demonstrate both the utility and the limitations of these teleconsultations. The question of the pertinence of this use of teleconsultation with respect to the criteria for consultations also remains to be addressed. Most of the studies performed over the last year have been quantitative and have aimed to demonstrate the strengths and weaknesses of telemedicine in oncology from a purely medical standpoint [7, 10–12]. In line with these studies, the aim of this work was to assess the feasibility of such consultations, but also to find answers to the ethical dimensions of medical questions. The originality of this work lies in the questions asked, based on an obligation linked to the pandemic to prevent (by precaution) vulnerable patients with cancers from being subjected to a risk of contamination with the virus through consultations in person. This led to reflections on the ways in which doctors had been led to change their practices and to use a technology that had not previously been part of those practices. During an internship at the ESME Sudria school, Lucas Huret (first author), a trainee engineer was asked to specify the conditions of use of this technology in practice, with the support of a technical reflection and comments from the professionals who had used it.

The various semi-directed interviews made it possible to identify difficulties encountered by the doctors of the oncology and supportive care departments of Foch Hospital in the use of telemedicine during the first wave of the COVID-19 epidemic. The constraints mentioned in international publications, particularly those relating to the social and ethnic origin of the patients [13], were not identified in this study. By contrast, as suggested by one doctor, another constraint concerned people living in underprivileged environments, who may not necessarily have access to the digital tools required for teleconsultation. Similarly, a foreigner with a poor command of the language of the country would have greater difficulty communicating with the doctor. If these factors were present but not taken into account, it would constitute a form of stigmatization [14]. In addition, it should be borne in mind that telemedicine renders virtual not only the consultations, but, above all, the patient-doctor relationship and the space in which this relationship develops [15]. According to the study “Cancer patients’ trust in their physician — a review”, the relationship of trust between the doctor and the patient is important and must be successful and durable: “*A trusting relationship between patient and physician resulted in facilitated communication and medical decision making, a decrease of patient fear, and better treatment adherence*” [11]. Dematerialization makes this imperative more complicated. Without this trust, despite the use of telemedicine to resolve the problem of remote consultation, patients may literally close up and consciously or unconsciously omit to communicate elements useful to the doctor in reflections on the management of the patient’s healthcare trajectory.

We were also able to distinguish certain important fears of doctors with respect to telemedicine in oncology. One of these fears stems from the fact that some of the software used for telecommunication, such as *Teams* and *Skype*, originates from GAFA (Google, Apple, Facebook, Amazon) [16, 17], also known as GAFAM (Google, Apple, Facebook, Amazon, Microsoft) [16], and is not considered secure. This group of North-American companies provide software housed by servers on European territory but belonging to private American entities [18]. In the context of international competition, it is important to bear in mind that data are becoming an asset driving national growth (https://www.numerique.gouv.fr/actualites/la-revolution-de-ladonnee-au-service-de-la-croissance-le-rapport-du-groupe-de-travailfranco-britannique/). In France and the rest of the European Union, health data are protected by the GDPR (general data protection regulation), but this housing of the data on private servers nevertheless poses an ethical and legal problem in terms of the ownership of French health data, an issue that is, of course, not specific to oncology [19]. As pointed out by one of the doctors interviewed *“We are handing the health of the French to GAFAM.”* However, we were surprised to find that this doctor, the only one to have considered the question of where the data are stored, had not thought about (or at least did not mention) the security of telecommunications networks, despite the fact that several secure networks already exist. Indeed, “ROSeS” (*Réseau Optique Sécurisé pour l’eSanté*; Secure Optical Network for e-Health) is a high-throughput secure network available to the adherents of the SESAN structure (https://www.sesan.fr/services/roses). However, the COVID-19 health crisis prompted our doctors to act quickly, ignoring this security aspect when considering the benefit/risk ratios of the possible solutions. The lack of time available for profound reflection and the sudden nature of the use of a new technology may explain the lack of questions raised by the doctors about the security of the network used. However, as we did not pose this question directly in our interview grid, we were unable to evaluate the opinions of the doctors questioned concerning this point. Nevertheless, questions about the security of health networks have already been raised in other studies, and some answers are available. These issues remain important in telemedicine. In oncology, as elsewhere, healthcare professionals should, therefore, continue to reflect on this strategic issue of importance for both the French health system and the economy of the country. This point also highlights the difficulties that may sometimes be encountered when applying GDPR conditions. Technical solutions that already exist (ROSeS, *Messagerie Sécurisée de Santé* (https://esante.gouv.fr/securite/messageries-de-sante-mssante), etc.) or could be envisaged should be generalized in the development of telemedicine tools (https://documentation.ehesp.fr/index.php?lvl=notice_display&id=333725).

One of the fears raised in our interviews was purely legal and related to issues of legal responsibility. Indeed, in the case of the non-detection of a recurrence of cancer or of late management, which can be a source of complaints about decreasing chances of survival [20], who is responsible? Penal responsibility is always engaged, but civil responsibility depends on the form of exercise: public or private. These questions are undoubtedly less often raised in France than in the United States, due to the ease of access to care. According to a study performed by the French National Cancer Institut (INCa) on a shared directory of healthcare professionals, there are between 1.73 and 2.90 oncologists per 100,000 inhabitants in the Ile-de-France region, versus a mean national value of 1.73 per 100,000 inhabitants (https://www.e-cancer.fr/Professionnels-de-sante/Les-chiffres-ducancer-en-France/Equipements-et-dispositifs-de-prise-en-charge). Medicolegal questions may, thus, have a lesser impact in Ile-de-France, which is particularly well endowed with oncologists, and their impact may vary according to the environment, potentially accounting for their poor representation in the responses to the questions posed during our interviews. Telemedicine is still a source of many questions, which remain to be resolved [21], many of which lie well beyond the realm of oncology, as pointed out by Sylvie Morel at the Congress of the Swiss Association of Sociology in September 2019, *“This emergence of emergency telemedicine is accompanied by new questions relating to the modes of use of these new ICTs and their effects on the conditions of the exercise, working practices, professional cultures and identities, relationships between healthcare professionals and the movement of professional boundaries”* [22].

These various empirical and bibliographic elements demonstrate the need for training in telemedicine for healthcare professionals generally, not just in oncology. The integration of academic training in telemedicine directly into the curriculum of studies in health would make it possible to develop the needs of universities for reflections on the application of new technologies, such as telemedicine, with the collaborative participation of engineers and doctors. Professional training at the hospital might be more appropriate, with direct contact with the designers of these computer programs. The simultaneous use of both these forms of training would probably be the best option. One particular aim is the establishment of a true relationship with the patient, particularly as this new tool is likely to become indispensable in the future [12, 23, 24]. Professional training at the hospital would make it possible for doctors who have received little or no theoretical or practical teaching in telemedicine to acquire the necessary knowledge and skills now required more rapidly. Above all, this training would enable the developers of the software used in this context to obtain direct feedback on its use and a more precise vision of the real needs of healthcare professionals. However, before that, a more profound reflection in engineering [25] may be required, a reflection inspired by interfaces with doctors (in this case, oncologists) and engineers, as in this study. Unambiguous academic and professional training courses in telemedicine already exist for certain healthcare professionals, and there is a two-day training session for nurses organized by the training organization Orion Santé, and another organized by Pôle Formation de Santé (https://www.orionsante.fr/trouver-une-formation/telemecine, https://www.poleformation-sante.fr/formation/telemedecinetelesoins). The University of Bordeaux has established an interuniversity diploma (DIU) in telemedicine in collaboration with the universities of Besançon, Montpellier, Lilles, Nantes and Caen (http://www.diu-telemedecine.fr/). Furthermore, courses in telemedicine are being integrated into certain study programs, such as the university diploma (DU) in stroke emergencies of Sorbonne University and the DIU in gerontechnology at the same university (https://fc.sorbonne-universite.fr/nos-offres/du-urgences-avc/, https://fc.sorbonne-universite.fr/nos-offres/diu-gerontechnologiesante-et-autonomie/). Thus, training courses do exist, but they remain few in number and monodisciplinary. The French government has included telemedicine in a new project, “Ma Santé 2022”, but with no mention of any training in this new technology (https://solidaritessante.gouv.fr/IMG/pdf/ma_sante_2022_pages_vdef_.pdf). This lack of education shows that we have underestimated telemedicine as a tool. This article poses the question and proposes to increase training, according to the diseases encountered in diverse consultations, by developing criteria for access to telemedicine, for application outside the pandemic context, in particular.

Drawing a parallel with Canada, which has pioneered telemedicine [26], could provide us with a more global vision. Firstly, Canada has a history of telemedicine use [26, 27], whereas France, despite the legal recognition of this practice since the law reforming hospitals and relating to patients, health and territories (the HPST law) of July 22 2009 (https://solidarites-sante.gouv.fr/professionnels/gerer-unetablissement-de-sante-medico-social/financement/financement-desetablissements-de-sante-10795/financement-des-etablissements-desante-glossaire/article/loi-hpst-hopital-patients-sante-territoires), has only been in a position to use this technology effectively since 2018, with the publication of the decree of August 1 in supplementary clause 6 of the medical convention and the decree of September 13 (https://www.legifrance.gouv.fr/loda/article_lc/LEGIARTI000037439234/2018-08-11, https://www.legifrance.gouv.fr/loda/id/JORFTEXT000037399738/). This tool was, thus, deployed much later in France, but has been used for relatively similar reasons, such as ensuring equal access to care (https://www.has-sante.fr/jcms/c_2673715/fr/telemedecine) , the principal reason for its use in Canada (https://numerique.banq.qc.ca/patrimoine/details/52327/4179514, https://docplayer.fr/77533137-Telemedecine-journee-d-informationdepartementale-08-12-2017-delegation-departementale-hautevienne.html) . However, it was the COVID-19 crisis that demonstrated the utility of telemedicine in France and led to an extremely rapid expansion of its use [28]. This spread of the use of telemedicine has also amplified the dimension of patient quality of life, as we can see in the results, at least for oncology. It would be interesting to take into account the difficulties encountered by the Canadians and the solutions they have found during their experience. The “Implantation of telehealth and of its long-term use in Canada: a few lessons to be learned” study defined conditions for the use of telemedicine. These conditions were *“organizational support and governance; appropriate and recurrent funding; adequate technologies and technological environments; strategies for active and targeted communication with decision-making bodies and the public; continuous training and development; and assertive political leadership”* [29]. France covers a much smaller area than Canada, with territorial problems that are also likely to be less important, but, essentially, the same questions remain. The responses given in the article cited above are, therefore, pertinent, and address some of the concerns of the doctors questioned here. It is now our turn to think deeply about the application of telemedicine in France. Such reflections are already underway, as attested by the many articles and dissertations on this theme published in recent years (https://numerique.banq.qc.ca/patrimoine/details/52327/4179514, https://docplayer.fr/77533137-Telemedecine-journee-d-informationdepartementale-08-12-2017-delegation-departementale-hautevienne.html, [2, 18, 30, 31]).

Following on from these reflections, as explained above, it appears to us that several elements merit further consideration. One of these elements is the risk of favoring teleconsultation over consultation in person for financial reasons, given that teleconsultation entails fewer costs because there is no need for transport (ambulance, taxi, light health vehicle). This cost-reducing aspect merits closer study. In addition, as telemedicine could be considered to be a new form of medicine, it could require certification for its application, as we can see with the advent of professional and academic training courses, such as DIUs, and the inclusion of telemedicine courses in DUs (http://www.diu-telemedecine.fr/). For a technology of this type that is likely to continue to develop, an ethical reflection — concerning informed consent in particular, which may today appear vague in telemedicine, in terms of agreeing to teleconsultation on a specialist site or program, or knowing whether it is necessary to request consent before each teleconsultation — is inevitable [32]. The practice of dynamic consent could also benefit from telemedicine [33]. Indeed, a request for consent in videoconference, following an explanation of practices ahead of the performance of a medical act, could be envisaged. Finally, one little-mentioned element that is slowing the uptake of telemedicine is the overprotection of data in France, and, more specifically, the difficulty obtaining access to data [34]. The protection of health data is of the utmost importance, but the overproduction of these data limits the development of telemedicine, like many other medical or similar technologies. For example, we have seen, in particular, how difficult it has been to establish personal medical files [35] and the difficulties encountered in development of *“the personal digital space”* of the recent *“Ma Santé 2022”*reform (https://solidarites-sante.gouv.fr/actualites/presse/dossiers-depresse/article/dossier-de-presse-ma-sante-2022-un-engagementcollectif). For this reason, industry and academics must now face up to challenges to meet the expectations, whether conscious or otherwise, of the medical sector.

## Conclusions

The COVID-19 pandemic forced various doctors from the oncology and supportive care departments of Foch Hospital to use, with circumspection, a tool that had hitherto been little envisaged and even less used. The development and use of telemedicine from the first wave of the epidemic highlighted the indications and the precautions to be taken and revealed that this technique was a feasible solution for the follow-up of patients with cancer, both in the context of a crisis, and outside of this context.

Clearly, many gray zones concerning telemedicine remain to be cleared up, and there are, above all, questions concerning the capacity of technology to meet current needs, particularly in the context of cancer. In the near future, this technology could conceivable be developed for diagnosis, with is currently impracticable by teleconsultation, through the use of colored markers, for example, to highlight abnormalities [36], such as nodules. The utility of such a development is from the concern raised by one oncologist: *“I diagnose recurrences on the skin with nodules that appear… And, that, if you don’t examine your patient, you don’t pick it up.”* This comment, suggesting that there will always be patients needing classic clinical examination during consultations in person, led to our search for criteria making it possible to use telemedicine routinely, on an everyday basis. Telemedicine could, potentially, be used to establish a diagnosis remotely, but we are not there yet. It could also make it possible to use virtual reality, which is already used in certain practices, for educational purposes [37, 38]. This technology is promising, but we need to move forward carefully, keeping improvements in patient quality of life and survival at the heart of our reflections.

Hospital doctors have reacted well to the radical change linked to the performance of remote consultations. Telemedicine could herald a revolution in the management of cancer patients [39]. However, our study, which is qualitative in nature, shows that certain ideas must be tempered. Telemedicine has the potential to become an important tool in the medical arsenal, but has not yet been sufficiently explored to be considered a revolution, particularly in the field of cancer [40]. Indeed, it remains possible that the use of telemedicine has been introduced too early, potentially delaying care, and even leading to late diagnoses. Further technological and human efforts are still required for telemedicine to be “unanimously” accepted by medical professionals, even if there will probably always be some reluctance, as shown by the study performed by Elif Shanin et al. [10]. This technology establishes connections between three parties: doctors, patients and industry. Cooperation between these three parties appears to us to be as crucial as it is currently insufficient. The decision to use telemedicine should be taken by a trained doctor, in concertation with patients, who should indicate their preferences. Patients in a state of anxiety tend to pay insufficient attention to decisions of this type [7, 41]. Clearly explaining the implications and utility of telemedicine to patients would go some way to overcoming this problem.

Telemedicine is still at the developmental stage, but has already proved useful in the context of health crises. Telemedicine met many of the needs of the doctors of the oncology and supportive care departments of Foch Hospital during the COVID-19 pandemic. As shown by this study, the healthcare professionals were faced with sometimes very difficult questions concerning access to care, and telemedicine was identified as a means of overcoming these problems. With hindsight, it is in the interests of all concerned to evaluate the fairness, difficulties, adverse effects and benefits of this solution. It will, therefore, be important to reflect on this sudden change in practice, and the use of a tool outside its usual context. Efforts to develop and to deploy telemedicine remain in their infancy, and should be followed attentively.

## Data Availability

We confirm that the raw data can be obtained on demand, by contacting the corresponding author.
